# Pulmonary Embolism Triage: When to Do What

**DOI:** 10.14797/mdcvj.1394

**Published:** 2024-05-16

**Authors:** Kathryn M. Donovan

**Affiliations:** 1 Department of Cardiovascular Surgery, Houston Methodist, The Woodlands, Texas, US

**Keywords:** pulmonary embolism, cor pulmonale, right heart strain

## Abstract

Anticoagulation has been the standard therapy for treating pulmonary embolism. However, newly developed pharmacological and interventional treatment options have been shown to provide benefit for certain patient populations, depending on how they present. This column highlights the use of massive pulmonary embolism risk stratification in determining the presence of cor pulmonale and offers several key points to remember when caring for patients with a pulmonary embolism.

## Introduction

Pulmonary embolism has historically been treated with anticoagulation alone; however, with the emergence of new advancing pharmacological and interventional adjuncts, risk stratification and treatment modalities have become more complex. There is a litany of pulmonary embolism presentations that can sometimes make decision-making difficult. This Points to Remember column serves to guide individuals on the management of acute pulmonary embolism through the use of risk stratification. The first step in acute pulmonary embolism triage should be determining the presence or absence of cor pulmonale: dilation of the right ventricle with reduced systolic function secondary to increasing pulmonary pressures.^1,2,3^

The following offers several key points to remember when caring for patients with a pulmonary embolism.

## Case

A 70 year-old-male with no significant medical history was brought into the emergency room by Emergency Medical Services (EMS) after a syncopal episode. The patient was working out at home when he suddenly became short of breath and lost consciousness, as witnessed by his spouse. Upon EMS presentation, the patient was hypotensive (systolic blood pressure in the 70s), tachycardic (heart rate 120s) and in respiratory distress (SaO_2_ 80% on room air). Upon arrival to the emergency department, he was noted to be normotensive without requirement of vasopressors, but he continued to be dyspneic and was placed on bilevel positive airway pressure. A computed tomography (CT) pulmonary angiogram was conducted and showed a large saddle embolus and right ventricular/left ventricular ratio (RV:LV) > 1.

The patient was assessed by the vascular surgery team while in the CT scanner, given a bolus of heparin, and promptly taken to the operating room for pulmonary artery thrombectomy. The patient was removed from oxygen postoperatively and discharged the following day on oral anticoagulation. A follow-up echocardiogram was conducted 3 weeks postoperatively and showed normal RV size and systolic function.

## Points to Remember About Management of Pulmonary Embolism

- There are multiple avenues available when determining the presence or absence of cor pulmonale, but it is always best to stick to a systematic approach. Imaging, or CT evidence of right heart strain with elevated RV/LV ratio, can easily confirm suspicions. Other key data points for determining the presence of cor pulmonale include: history of present illness (HPI), physical exam, electrocardiographic findings of sinus tachycardia or new-onset atrial fibrillation, elevated cardiac biomarkers, and echocardiogram findings consistent with RV dilation or reduced RV systolic function.^4,5,6,7,8,9^
- Echocardiograms are useful for determining cor pulmonale when you question the presence of right heart strain, but it should be noted that the duration and accessibility of this study can often delay care in critically ill patients. If you have CT evidence of right heart strain, in combination with tachycardia or hypotension, an echocardiogram should not delay definitive PE care.^10,11^
- A good history, or story, can make all the difference. Patients with cor pulmonale often complain of shortness of breath, chest pain, and cyanosis of syncope. Regardless of imaging findings, a patient’s history may show signs of severe right heart dysfunction and can be warning signs of impending decline. Therefore, the HPI should be incorporated in risk stratification for compressive, individualized decision making.^5,6,7^
- Anticoagulation remains the initial treatment of choice for all PEs. When it comes to reperfusion therapy in the intermediate risk population, risk stratification along with highly individualized assessment should be used to determine benefit. High-risk pulmonary embolisms should be treated with reperfusion therapy to rapidly offload the right heart. The modality or reperfusion should be based on local expertise and resources.^1,2,12,13,14,15,16^
